# HN-CNN: A Heterogeneous Network Based on Convolutional Neural Network for m^7^ G Site Disease Association Prediction

**DOI:** 10.3389/fgene.2021.655284

**Published:** 2021-03-04

**Authors:** Lin Zhang, Jin Chen, Jiani Ma, Hui Liu

**Affiliations:** ^1^Engineering Research Center of Intelligent Control for Underground Space, Ministry of Education, China University of Mining and Technology, Xuzhou, China; ^2^School of Information and Control Engineering, China University of Mining and Technology, Xuzhou, China

**Keywords:** m^7^G sites, diseases, heterogeneous network, convolutional neural network, XGBoost

## Abstract

N^7^-methylguanosine (m^7^G) is a typical positively charged RNA modification, playing a vital role in transcriptional regulation. m^7^G can affect the biological processes of mRNA and tRNA and has associations with multiple diseases including cancers. Wet-lab experiments are cost and time ineffective for the identification of disease-related m^7^G sites. Thus, a heterogeneous network method based on Convolutional Neural Networks (HN-CNN) has been proposed to predict unknown associations between m^7^G sites and diseases. HN-CNN constructs a heterogeneous network with m^7^G site similarity, disease similarity, and disease-associated m^7^G sites to formulate features for m^7^G site-disease pairs. Next, a convolutional neural network (CNN) obtains multidimensional and irrelevant features prominently. Finally, XGBoost is adopted to predict the association between m^7^G sites and diseases. The performance of HN-CNN is compared with Naive Bayes (NB), Random Forest (RF), Support Vector Machine (SVM), as well as Gradient Boosting Decision Tree (GBDT) through 10-fold cross-validation. The average AUC of HN-CNN is 0.827, which is superior to others.

## Introduction

N^7^-methylguanosine (m^7^G) is one of the most abundant modifications present in tRNA, rRNA, and mRNA 5′cap and plays critical roles in regulating RNA processing, metabolism, and function (Malbec et al., 2019). As an essential post-transcriptional modification, m^7^G plays an essential role in gene expression, processing and metabolism, protein synthesis, transcription stability and other aspects (Pandolfini et al., 2019). m^7^G is often enriched in the 5′UTR region and AG-enriched contexts. The internal m^7^G modification is dynamically regulated under both H_2_O_2_ and heat shock treatments, with remarkable accumulations in CDS and 3′UTR regions and functions in promoting mRNA translation efficiency (Malbec et al., 2019). m^7^G_46_ methylation of specific tRNA is associated with human mutation and the corresponding yeast mutation, which is m^7^G modification at position 46 in tRNA. Reduced m^7^G_46_ modification causes a growth deficiency phenotype in yeast, which provides a potential mechanism for primordial dwarfism associated with this lesion (Shaheen et al., 2015).

Munns et al. (1985) concluded that a specific autoimmune disorder is associated with the presence of anti-m^7^G autoantibodies in 50 patients’ cases. Bradrick (2017) found that mosquito-borne flaviviruses are important human pathogens, and m^7^G of the 5′cap structure is essential for infection. Lin et al. (2018) developed m^7^G methylated tRNA immunoprecipitation sequencing (MeRIP-seq) and tRNA reduction and cleavage sequencing (TRAC-seq) to conform that Mettl1-mediated tRNA m^7^G modification is essential for the proper expression of neural lineage genes. m^7^G methyltransferase complex METTL1/WDR4 causes primordial dwarfism and brain malformation. Thus, m^7^G sites and human diseases may show associations (Enroth et al., 2019). The study of disease-associated m^7^G may reveal the pathogenesis of the disease.

However, there is still a lack of systematic research on RNA modification due to technical limitations. Few studies have systematically explored the association between m^7^G sites and diseases. It is laborious and expensive to find disease-related m^7^G sites by wet-lab experiments. Recently, more and more artificial intelligence methods have been applied in the analysis of biological data. It can be regarded as a classification issue for disease-related m^7^G sites prediction, where the known association is denoted as 1, 0 otherwise. Some classical classifiers can be used to solve this problem, such as Naive Bayesian (NB), Support Vector Machine (SVM), Random Forest (RF), Gradient Boosting Decision Tree (GBDT), and Matrix Factorization (MF). With Bayes theorem, NB is proposed, which has a strong bias for linearity (Ting and Zheng, 2003). The prediction accuracy decreases dramatically in nonlinear scenarios. SVM is known to be suitable in small sample and nonlinear scenarios (Chang and Lin, 2011), which depends on the kernel to map data to a high-dimensional space. The data about disease-related m^7^G sites are high sparsity, so it is not easy to find the appropriate kernel. RF is an essential method in machine learning and has been widely used in many fields (Ham et al., 2005). However, it is not easy to obtain high precision and generalization performance simultaneously. GBDT is suitable for regression analysis, but the computation load is too high (Rao et al., 2019). Consistent with RF, it is also not suitable for sparse data. MF is the classic model of recommendation system (Lee and Seung, 1999). The low-rank matrix can be used to predict the association between m^7^G sites and diseases. But the higher the requirement of a low-rank matrix, the longer the training time.

In this paper, a deep learning framework based on heterogeneous networks and convolutional neural networks is proposed to find disease-associated m^7^G sites. The site-site similarities were calculated according to the chemical structure of m^7^G site, and the disease–disease similarities were achieved by miRNAs based on induced disease sets. Simultaneously, the known associations between the m^7^G site and the disease were incorporated into the heterogeneous network. Then, the convolutional neural network (CNN) was then adopted to extract multidimensional feature, making full use of the sparse data. Finally, XGBoost was used to predict the associations between m^7^G sites and various diseases.

## Materials and Methods

### Datasets

m7GDiseaseDB is an m^7^G-disease association database by taking 1218 disease-associated genetic variants as a bridge, which may lead to gain/loss of the m^7^G sites, with implications for disease pathogenesis involving m^7^G RNA methylation (Song et al., 2020). Among them, 768 associations between 741 m^7^G sites and 177 diseases were extracted via 741 variants with high confidence levels in m7GDiseaseDB. Specifically, the genomic locations, host genes of those sites were also included for further feature calculation.

In the mathematical view, let **R** ∈ ℝ^M×N^ be the association matrix consisting of *M* sites *S* = {*s*_1_,*s*_2_,⋯,*s*_M_} and *N* diseases *D* = {*d*_1_,*d*_2_,⋯,*d*_N_}. If there is an association between m^7^G site *s*_*i*_ and disease *d*_*j*_, *R*_*ij*_ is 1, 0 otherwise.

### Heterogeneous Network Based on Convolutional Neural Network

**FIGURE 1:**
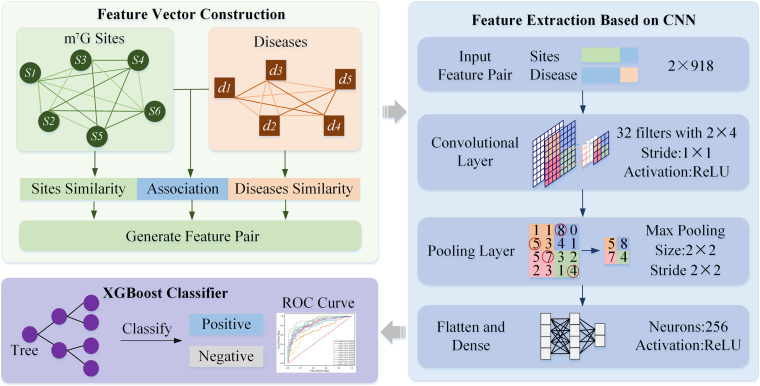
Framework of HN-CNN. “Feature Vector Construction” is a heterogeneous network based on feature extraction, which is constructed with similarities and known m7G-disease association. “Feature Extraction Based on CNN” is a CNN-based feature extraction followed by XGBoost. In “XGBoost Classifier,” XGBoost predicts the candidate samples, which chooses the regression classification tree as a base learner.

Figure 1 illustrates the framework HN-CNN. A heterogeneous network was constructed with site-site similarity, disease–disease similarity and the known m^7^G-disease associations to generate feature pairs. Then, each feature pair was transformed into a vector with high-dimensional hidden information by CNN. XGBoost predicts the candidate samples lastly, which chooses the regression classification tree as a base learner.

## Feature Vector Construction

Chemical properties of m^7^G sites were utilized to depict the m^7^G feature just as previously described in similar work (Chen et al., 2019). Based on the chemical features of m^7^G sites, the site similarities were calculated by Jaccard coefficient which is defined as Equation as (1):

(1) $$\mathrm{Jaccard}\mathrm{similarity}=\frac{\left|\text{A}\cap \text{B}\right|}{\left|\text{A}\cup \text{B}\right|}=\frac{\left|\text{A}\cap \text{B}\right|}{\left|A\right|+\left|B\right|-\left|\text{A}\cap \text{B}\right|}$$

where ***A*** and ***B*** represent the chemical feature of two sites.

**FIGURE 2:**
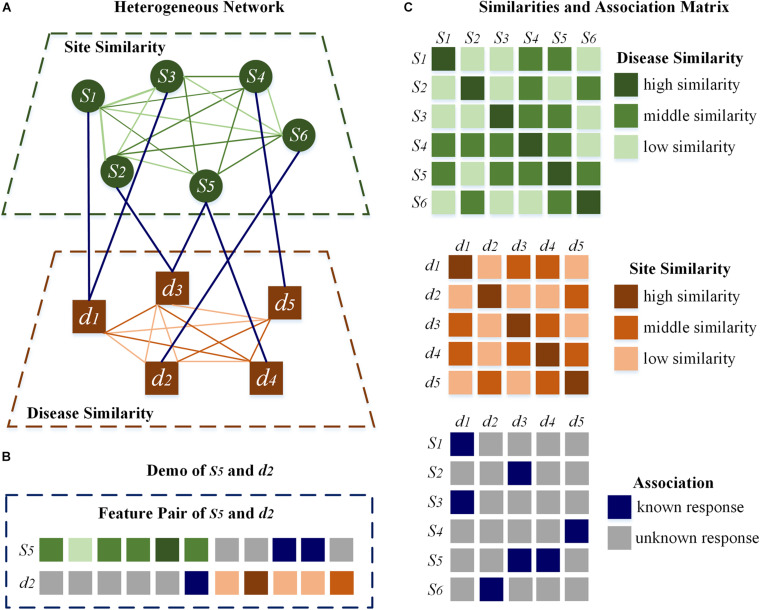
Heterogeneous network and feature pair. **(A)** The heterogeneous network. **(B)** The demo of *s*_5_ and *d*_2_. **(C)** The related matrixes directly.

In addition, the disease–disease similarity is calculated by DisSetSim (Hu et al., 2017), which is an online system for calculating similarity with diseases names and open source databases. Disease-related genes, functional annotation of genes and the gene functional network of human are involved in calculating disease–disease similarity. Heterogeneous network adopts site-site similarity, disease–disease similarity, combined with the known association between m^7^G sites and diseases, shown directly in Figure 2A.

HN-CNN pays more attention to the latent description of associations of m^7^G sites and diseases. Similarities and association are included in the heterogeneous network. Taking *s*_5_ and *d*_2_ in Figure 2B as an example, vector related to *s*_5_ is selected from the association matrix and site–site similarity, which is different from other sites. Vector related to *d*_2_ is selected from disease–disease similarity and the association matrix to form the vector of *d*_2_. Those two vectors combine to form the feature pair about *s*_5_ and *d*_2_, and each pair is unique. Therefore, the feature pair retains the commonness and the characteristics. Commonness means that the vector representing the same site or disease is invariant. Characteristics means the combination of site-disease is unique, which is different from any other feature pairs. Finally, the feature pair, which is shown in Figure 2B, is the connection between heterogeneous network and CNN.

## Feature Extraction Based on CNN

Convolutional neural network (CNN) has a deep learning structure, which can mine hidden information. It is superior to the single network in terms of feature extraction and model fitting (Shin et al., 2016). The input layer becomes a multidimensional characteristic surface through the convolutional layer, and the propagation mode between the convolutional layers is shown in Equation (2). Then, features are mapped by pooling, and maximum pooling is shown in Equation (3). Finally, the selected features are flattened to form the final feature vectors:

(2) Hjl=σ(∑i=1NHil-1kijl*+bjl)

where $H_{j}^{l}$ is the *j*-th feature map of the *i*-th layer, *N* is the number of the *i*-th layer’s kernels, $k_{ij}^{l}$ is the *j*-th element in the *i*-th convolution kernel at the *l* layer, $b_{j}^{l}$ is the bias parameters, σ is the activation function:

(3) $${\text{Pooling}}_{j}^{l}=\max_{p\times q}\left({\text{H}}_{j}^{l}\right)$$

Where *max*_*p×q*_ chooses the maximum from ${\text{H}}_{j}^{l}$ with the *p*×*q*-size pooling. The ${\text{Pooling}}_{j}^{l}$ is the *j*-th pooling vector in the *l*-th layer.

Although the feature pairs were achieved in the previous section, the data is sparse with little information. The convolutional layer comprises multiple convolution kernels, which mine different characteristics of feature pairs. Therefore, the generated feature pairs are extracted by CNN. After that, feature vectors are formed, which contain not only various but also different information. In this paper, the associations of adjacent data in feature pairs are weak, so the convolution step size is set as 1 to make full use of each known data and mine each data’s hidden information. If the step size becomes bigger, some information will be ignored. The convolution kernel’s width was set as 2 to explore the association between m^7^G sites and diseases. To extract more dimensional information and mine the diverse relationships in feature pairs, the more convolution kernels are used, the better performance we have. However, the more computing resources and the longer the computation time are needed with too many kernels, along with the higher repetition rate. Considering high sparsity between the data, such as the sparsity of disease–disease similarity is 72.78%, the number of convolution kernels is set to 32. Meanwhile, the prediction accuracy is the best by experiment. If the number of convolution kernels is reduced, the accuracy will be decreased for mining the information of feature pairs deficiently. When the number of convolution kernels is increased, the accuracy is also decreased for repeated or useless features.

Then, the data are passed into the pooling layer. The pooling layer can reduce the input information dimension, keep the characteristic invariance, select the primary information, and reduce the redundancy information. In this paper, the size of maximum pooling is 2 × 2. Length 2 can screen out the data with prominent characteristics between sites and diseases; width 2 can effectively remove the duplicate data and screen out the critical information that has been expanded to the higher dimension.

Finally, feature pairs have been processed into vectors containing various kinds of information, but those vectors contain a large amount of information, with many types. The pooled vectors are compressed by full connection to integrate the feature data. The final feature vectors $V=\left\{v_{1}^{d},v_{2}^{d},\cdots v_{\text{n}}^{d}\right\}$ are formed, where *n* is the number of known associations, and *d* is the number of neurons in the full connection layer. In this paper, *d* is set to 256. When *d* is less than 256, the performance dramatically decreases due to less information in ***V***. The performance also decreased due to too much or even useless information in ***V***. ***V*** contains categorical information, optimizing by cross-entropy, to make ***V*** highly relevant to the original information, and ***V*** is used by subsequent classifiers.

## XGBoost Classifier

XGBoost classifier is adopted to predict associations between m^7^G site and disease. It retains the feature information better and weakens the influence of parameters on final accuracy. As an integrated learning algorithm that optimizes distributed gradient enhancement, XGBoost has good performance in generalization by regulation and second-order Taylor expansions (Torlay et al., 2017). In this article, the regression classification tree is chosen as a base learner, whose input is ***V***, and output is shown in Equation (4):

(4) $${\hat{y}}_{i}=\sum_{k=1}^{K}f_{k}\left(v_{i}\right),f_{k}\in E$$

where ${\hat{y}}_{i}$ is the result, *v*_*i*_ is the *i*-th vector in eigenvector ***V***, *f*_*k*_ is the *k*-th decision tree, *K* is the number of leaf nodes, and ***E*** is the set of classification regression trees. The optimized objective function for XGBoost is shown in Equation (5):

(5) $$L=\sum_{i=1}^{n}l\left({\hat{y}}_{i},y_{i}\right)+\sum_{k=1}^{K}\Omega \left(f_{k}\right)$$

where *y*_*i*_ is the ground truth, and $l\left({\hat{y}}_{i},y_{i}\right)$ is binary cross-entropy loss and shown in Equation (6):

(6) $$l\left({\hat{y}}_{i},y_{i}\right)=y_{i}\ln\left(1+e^{-{\hat{y}}_{i}}\right)+\left(1-y_{i}\right)\ln\left(1+e^{{\hat{y}}_{i}}\right)$$

Ω(*f*_*k*_) is regularization to prevent overfitting and enhance generalization ability. Ω(*f*_*k*_) is shown in Equation (7):

(7) $$\Omega \left(f\right)=\gamma T+\frac{1}{2}\lambda {∥w∥}^{2}$$

where *γ* is the complexity cost by adding new leaf nodes. *T* is the number of leaves in a tree. ||*w*||^2^ is the sum of the square of each leaf node. *λ* is the regularization coefficient about the L2 norm ||*w*||^2^.

There are several hyperparameters in XGBoost such as the complexity cost of adding new leaf nodes *γ* and the regularization coefficient *λ*. To achieve better AUCs, cross validation is inlaid into XGBoost to find the best parameters with *γ*∈{0,0.2,0.4,0.6,0.8,1} and *λ*∈{0, 0.01, 0.001}. Meanwhile, early stopping is adopted to avoid overfitting.

## Results

In this paper, HN-CNN is proposed to predict the association between m^7^G sites and diseases, and the performance is evaluated by 10-fold cross-validation. The original correlation matrix only marks the known relationship of m^7^G sites and diseases that can be considered positive, but the unknown does not mean negative. Thus, the same number of the negative is selected from unknown data randomly, and both the positive and the negative constitute the dataset. The set is divided into 10 parts on average, among which nine parts are used for training and the remaining 1 part for testing. The above operation should be repeated 10 times and the AUC should be recorded every time. It should be noted that the test set cannot be repeated in 10 training sets. After 10-folds, the average of 10 AUCs is the final result.

### Evaluation Metrics

HN-CNN predicts the positive probability of association between m^7^G sites and diseases. A threshold *θ* is needed when validation. If the probability is more prominent than *θ*, the sample is considered as positive. On the other hand, it is identified as negative. True positive rates (TPR) and false positive rates (FPR) are calculated according to the prediction and the truth [Equations (8) and (9)] (Hanczar et al., 2010):

(8) $$TPR=\frac{TP}{TP+FN}$$

(9) $$FPR=\frac{FP}{TN+FP}$$

where *TP* is true positive, *FP* is false positive, *TN* is true negative, and *FN* is false negative. If *θ* changes, *TPR* and *FPR* will also change. The receiver operating characteristic (ROC) curve is drawn with different *TPR*s and *FPR*s (Moses et al., 1993). ROC curve can display the performance of the model intuitively, but it cannot compare models accurately. The area under the ROC curve (AUC) can be used to evaluate the performance of classifier, which ranges from 0 to 1. The more AUC is close to 1, the better performance the classifier has (Fawcett, 2006). So, we choose the ROC curve and AUC to measure the models.

The $\bar{AUC}$ is the mean of *m* runs of 10-fold cross-validation, which is calculated by Equation (10):

(10) $$\bar{AUC}=\frac{1}{m}\sum_{j=1}^{m}\left(\frac{1}{10}\sum_{i=1}^{10}AUC_{i}\right)$$

where *m* is the number of experiments, *AUC*_*i*_ is the *i*-th AUC in 10-fold cross-validation. In this paper, *m* = 10.

### Comparison With Other Methods

**FIGURE 3:**
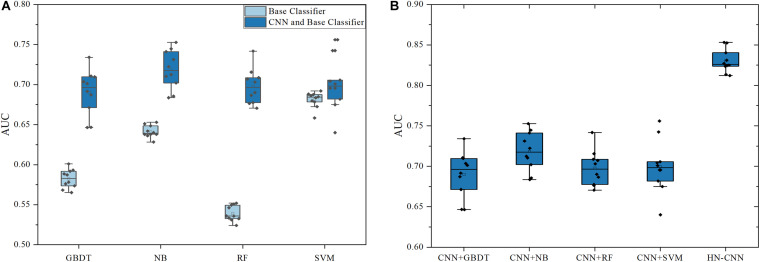
The AUCs of HN-CNN and other methods. **(A)** The AUCs of base classifiers with/without CNN. **(B)** The AUCs of HN-CNN and base classifiers with CNN.

To verify the advantages of CNN in extracting features, features that are not processed by CNN were compared with the features processed by CNN, which are classified with base classifiers such as GBDT, NB, SVM and RF. The result is shown in Figure 3A. The ordinate in the figure is the result of 10-fold cross verification, which is the average AUC. All average AUCs are calculated by 10-times of 10-fold cross-validation. The legends “Base Classifier” and “CNN and Base Classifier” are distinguished by whether the feature pair has been processed by CNN. “CNN and Base Classifier” means that feature pairs are processed with CNN, but the models of “Base Classifier” are not, which put feature pairs into classifiers directly.

According to the results in Figure 3A, it can be analyzed that the prediction accuracy is significantly improved after CNN extracts the feature with the same parameters and classifiers, which is the most obvious in the RF classifier. Without CNN, the mean AUC is 0.539 by RF. However, the average AUC is 0.698 with CNN, which increased by about 0.16. Besides, it is observed in Figure 3A that only the base classifiers without CNN have a greater impact on the prediction results. The average AUC directly predicted by SVM is 0.681, which is about 0.14 higher than that of RF. Classifiers with CNN improve the prediction effect and reduce the gap between classifiers. Therefore, CNN can effectively mine hidden data and improve classification accuracy.

The XGBoost was chosen as the final classifier for two reasons. XGBoost is an integrated machine learning algorithm based on decision trees, and its generalization performance is better than a single classifier. In other words, XGBoost finds the optimal solution within a fixed range of parameters. The results of XGBoost and other methods are shown in Figure 3B. CNN+GBDT in X-coordinate means that the features are extracted by CNN and classified by GBDT, and so on. The ordinate is the average AUC of 10-fold cross-validation. It can be analyzed that XGBoost is superior to the base classifiers. The average AUC of HN-CNN is 0.830, which is 0.111 higher than CNN+NB. Therefore, HN-CNN has the advantage in feature extraction and classification, which greatly improves the prediction accuracy.

### Case Study

The number of known associations is much less than the unknown, which can also be interpreted as the positive is much less than the negative. To weaken the influence of the negative, negative samples equal to the number of positive samples were selected randomly. The highest test accuracy in the 10-fold cross-validation was selected as the final prediction model, which predicts the positive probability of all unknown samples. We selected five of the top 20 to analyze and show the results in Table 1. R. analyzes the related genes with GO based on “clusterProfiler” (Yu et al., 2012). Among the results, CC is short for cellular component, MF is the molecular function, and BP is the biological process. Each gene description is described by *p*-value. If the *p* value is close to 0, the gene description is more obvious.

**TABLE 1:** Case study.

Disease	Gene	GO	*p*-value	Gene description
Combined oxidative phosphorylation deficiency	FOXRED1	BP	1.03E-04	Mitochondrial respiratory chain complex assembly
Xeroderma pigmentosum	EVC	BP	2.82E-04	Cartilage development
		BP	1.26E-03	Connective tissue development
Moyamoya disease	TPI1	MF	7.09E-04	Isomerase activity
Joubert syndrome	DNAJC5	BP	2.30E-04	Synaptic vesicle exocytosis
		BP	2.98E-04	Synaptic vesicle cycle
		BP	5.08E-04	Vesicle-mediated transport in synapse
		BP	1.23E-03	Neurotransmitter secretion
		BP	1.23E-03	Signal release from synapse
Brody myopathy	PET117	BP	1.03E-04	Mitochondrial respiratory chain complex assembly

Combined oxidative phosphorylation deficiency is caused by homozygous or compound heterozygous mutations in the ELAC2 gene, which is a mitochondrial tRNA processing gene (Haack et al., 2013). FOXRED1 can cause complex I deficiency and effect protein function (Calvo et al., 2010). Mitochondrial respiratory chain complex assembly mainly causes mitochondrial diseases (Deutschmann et al., 2014). There is a high correlation between disease and FOXRED1, in line with the laws of biology.

Xeroderma pigmentosum is a rare genetic disease characterized by extreme photosensitivity, resulting in a higher incidence of cutaneous tumors (Cleaver et al., 1999). EVC is essential for cartilage development (Pacheco et al., 2012). The *p*-value of connective tissue development is 1.26E-03, whose mutations contribute to tumor formation.

Moyamoya disease is a chronic, occlusive cerebrovascular disease with unknown etiology characterized by bilateral steno-occlusive changes at the terminal portion of the internal carotid artery and an abnormal vascular network base of the brain (Sakurai et al., 2004). Moyamoya disease is associated with various diseases, like atherosclerosis, autoimmune diseases, Down syndrome. TPI1 is a crucial enzyme in carbohydrate metabolism, negatively associated with tumor size (Jiang et al., 2017). Therefore, TPI1 may inhibit the size of tumors and induce Moyamoya disease.

Inheritance of Joubert syndrome is autosomal and recessive, which is characterized by hypoplasia of the cerebellar vermis (Kendall et al., 1990; Lee et al., 2012). DNAJC5 encodes the cysteine string protein, which is a presynaptic protein implicated in neurodegeneration (Cadieux-Dion et al., 2013). It causes autosomal dominant Kufs disease (Jarrett et al., 2018). One of Kufs’ phenotypes is generalized tonic-clonic seizures, which is similar to related disorders of Joubert syndrome (Chance et al., 1999; Josephson et al., 2001).

Brody myopathy is a rare muscle disorder characterized by exercise-induced impairment of muscle relaxation and stiffness (Odermatt et al., 2000). Pet117 is shown to reside in the mitochondrial matrix, associated with the inner membrane (Taylor et al., 2017). Its gene description hence mitochondrial respiratory efficiency, which is mitochondrial respiratory chain complex assembly (Cogliati et al., 2013). So, it may be further manifested as Brody myopathy symptoms.

## Discussion and Conclusion

It is efficient and time-saving to predict the association between m^7^G sites and diseases. HN-CNN integrates diverse information through heterogeneous networks. It adopts CNN to help extract latent relationships in feature pairs, which focuses on personalized associations between m^7^G sites and diseases. At last, XGBoost is used to classify whether there exists association with more generalization. In the 10-fold cross-validation, HN-CNN gets better results than the other methods. The predicted results are analyzed through R to show better demonstrated the reliability of the experimental method in case study. In the future, the data will be updated, and the sparsity will be reduced. HN-CNN will obtain better prediction results in the association prediction due to the amount of data.

## Data Availability Statement

The original contributions presented in the study are included in the article/Supplementary Material, further inquiries can be directed to the corresponding author/s.

## Author Contributions

JC and LZ reviewed the resources, wrote the manuscript, and revised the manuscript. JM provided the data and revised the manuscript. HL took the lead in the work and revised the manuscript. All authors contributed to the article and approved the submitted version.

## Conflict of Interest

The authors declare that the research was conducted in the absence of any commercial or financial relationships that could be construed as a potential conflict of interest.

## Supplementary Material

Supplementary Table 1 The top 50 of most likely site-disease associations.
