# Supplementary material for: HN-CNN: A Heterogeneous Network Based on Convolutional Neural Network for m7 G Site Disease Association Prediction
Source: Front Genet. 2021 Mar 4;12:655284. doi: 10.3389/fgene.2021.655284 (PMC7970120; doi:10.3389/fgene.2021.655284)
Supplement: Supplementary Table 1 — The top 50 of most likely site-disease associations. [file Table_1.DOCX]

Supplementary Material

# Supplementary Figures and Tables

| **Disease DOID** | **Disease Name** | **Gene** | **Rank** | **GO** | **pvalue** | **Gene Description** |
| --- | --- | --- | --- | --- | --- | --- |
| DOID:0060286 | Combined oxidative phosphorylation deficiency | FOXRED1 | 1 | BP | 1.03E-04 | mitochondrial respiratory chain complex assembly |
| DOID:9119 | Acute myeloid leukemia | KIF1B | 2 | CC | 1.38E-04 | cytoplasmic region |
|  |  |  |  | CC | 3.58E-04 | plasma membrane bounded cell projection cytoplasm |
| DOID:12549 | Hepatitis A | FGFR3 | 3 | BP | 1.35E-04 | chondrocyte differentiation |
|  |  |  |  | BP | 2.82E-04 | cartilage development |
|  |  |  |  | BP | 6.13E-04 | skeletal system morphogenesis |
|  |  |  |  | BP | 6.42E-04 | extrinsic apoptotic signaling pathway |
|  |  |  |  | BP | 1.26E-03 | connective tissue development |
|  |  |  |  | BP | 2.00E-03 | developmental maturation |
| DOID:13099 | Moyamoya disease | FGFR3 | 4 | BP | 1.35E-04 | chondrocyte differentiation |
|  |  |  |  | BP | 2.82E-04 | cartilage development |
|  |  |  |  | BP | 6.13E-04 | skeletal system morphogenesis |
|  |  |  |  | BP | 6.42E-04 | extrinsic apoptotic signaling pathway |
|  |  |  |  | BP | 1.26E-03 | connective tissue development |
|  |  |  |  | BP | 2.00E-03 | developmental maturation |
| DOID:0050427 | Xeroderma pigmentosum | EVC | 5 | BP | 2.82E-04 | cartilage development |
|  |  |  |  | BP | 1.26E-03 | connective tissue development |
| DOID:3883 | Lynch syndrome | ZIC2 | 6 | Not Found | | |
| DOID:3883 | Lynch syndrome | CP;HPS3 | 7 | Not Found | | |
| DOID:13099 | Moyamoya disease | TPI1 | 8 | MF | 7.09E-04 | isomerase activity |
| DOID:1925 | Coffin-Siris syndrome | PRKCSH | 9 | MF | 2.96E-05 | ion channel binding |
| DOID:1925 | Coffin-Siris syndrome | PRKCSH | 10 | MF | 2.96E-05 | ion channel binding |
| DOID:0050777 | Joubert syndrome | DNAJC5 | 11 | BP | 2.30E-04 | synaptic vesicle exocytosis |
|  |  |  |  | BP | 2.98E-04 | synaptic vesicle cycle |
|  |  |  |  | BP | 5.08E-04 | vesicle-mediated transport in synapse |
|  |  |  |  | BP | 1.23E-03 | neurotransmitter secretion |
|  |  |  |  | BP | 1.23E-03 | signal release from synapse |
| DOID:0050777 | Joubert syndrome | FGFR3 | 12 | BP | 1.35E-04 | chondrocyte differentiation |
|  |  |  |  | BP | 2.82E-04 | cartilage development |
|  |  |  |  | BP | 6.13E-04 | skeletal system morphogenesis |
|  |  |  |  | BP | 6.42E-04 | extrinsic apoptotic signaling pathway |
|  |  |  |  | BP | 1.26E-03 | connective tissue development |
|  |  |  |  | BP | 2.00E-03 | developmental maturation |
| DOID:0050692 | Brody myopathy | ZIC2 | 13 | Not Found | | |
| DOID:0050692 | Brody myopathy | WDR36 | 14 | Not Found | | |
| DOID:552 | Pneumonia | DEPTOR | 15 | BP | 5.90E-04 | regulation of apoptotic signaling pathway |
|  |  |  |  | BP | 6.42E-04 | extrinsic apoptotic signaling pathway |
|  |  |  |  | BP | 8.73E-04 | regulation of extrinsic apoptotic signaling pathway |
| DOID:0050692 | Brody myopathy | PET117 | 16 | BP | 1.03E-04 | mitochondrial respiratory chain complex assembly |
| DOID:0050884 | Triosephosphate isomerase deficiency | DNAJC5 | 17 | BP | 2.30E-04 | synaptic vesicle exocytosis |
|  |  |  |  | BP | 2.98E-04 | synaptic vesicle cycle |
|  |  |  |  | BP | 5.08E-04 | vesicle-mediated transport in synapse |
|  |  |  |  | BP | 1.23E-03 | neurotransmitter secretion |
|  |  |  |  | BP | 1.23E-03 | signal release from synapse |
| DOID:3883 | Lynch syndrome | RAB18 | 18 | BP | 2.79E-04 | endomembrane system organization |
| DOID:0060286 | Combined oxidative phosphorylation deficiency | NDUFS8 | 19 | BP | 2.20E-04 | NADH dehydrogenase complex assembly |
| DOID:13099 | Moyamoya disease | PET117 | 20 | BP | 1.03E-04 | mitochondrial respiratory chain complex assembly |
| DOID:0050777 | Joubert syndrome | CDC6 | 21 | Not Found | | |
| DOID:13189 | Gout | NPR2 | 22 | BP | 4.94E-05 | heart contraction |
|  |  |  |  | BP | 5.91E-05 | cardiac conduction |
|  |  |  |  | BP | 6.29E-05 | heart process |
|  |  |  |  | BP | 1.68E-04 | regulation of heart contraction |
|  |  |  |  | BP | 3.45E-04 | regulation of cardiac conduction |
|  |  |  |  | BP | 3.59E-04 | multicellular organismal signaling |
|  |  |  |  | BP | 4.68E-04 | regulation of blood circulation |
|  |  |  |  | BP | 2.00E-03 | developmental maturation |
| DOID:0050692 | Brody myopathy | TCAP | 23 | BP | 3.24E-08 | actin filament-based movement |
|  |  |  |  | BP | 9.00E-08 | actin-mediated cell contraction |
|  |  |  |  | BP | 6.84E-06 | pattern specification process |
|  |  |  |  | BP | 4.33E-05 | cardiac muscle contraction |
|  |  |  |  | BP | 4.94E-05 | heart contraction |
|  |  |  |  | BP | 6.29E-05 | heart process |
|  |  |  |  | BP | 6.37E-05 | anterior/posterior pattern specification |
|  |  |  |  | CC | 2.67E-04 | Z disc |
|  |  |  |  | MF | 2.96E-05 | ion channel binding |
| DOID:1925 | Coffin-Siris syndrome | CDC6 | 24 | Not Found | | |
| DOID:0050692 | Brody myopathy | DNAJC5 | 25 | BP | 2.30E-04 | synaptic vesicle exocytosis |
|  |  |  |  | BP | 2.98E-04 | synaptic vesicle cycle |
|  |  |  |  | BP | 5.08E-04 | vesicle-mediated transport in synapse |
|  |  |  |  | BP | 1.23E-03 | neurotransmitter secretion |
|  |  |  |  | BP | 1.23E-03 | signal release from synapse |
| DOID:0050692 | Brody myopathy | ALOX12B | 26 | BP | 2.48E-04 | ceramide biosynthetic process |
|  |  |  |  | BP | 1.13E-03 | ceramide metabolic process |
|  |  |  |  | BP | 1.45E-03 | sphingolipid biosynthetic process |
|  |  |  |  | MF | 7.09E-04 | isomerase activity |
| DOID:0050692 | Brody myopathy | SYNE2 | 27 | BP | 3.24E-08 | actin filament-based movement |
|  |  |  |  | BP | 5.06E-04 | gliogenesis |
|  |  |  |  | BP | 9.23E-04 | cerebral cortex cell migration |
|  |  |  |  | BP | 2.02E-03 | telencephalon cell migration |
|  |  |  |  | CC | 2.67E-04 | Z disc |
|  |  |  |  | CC | 4.04E-04 | I band |
|  |  |  |  | CC | 4.46E-04 | sarcoplasm |
|  |  |  |  | CC | 7.46E-04 | sarcoplasmic reticulum membrane |
|  |  |  |  | CC | 2.32E-03 | sarcomere |
| DOID:12549 | Hepatitis A | DNAJC5 | 28 | BP | 2.30E-04 | synaptic vesicle exocytosis |
|  |  |  |  | BP | 2.98E-04 | synaptic vesicle cycle |
|  |  |  |  | BP | 5.08E-04 | vesicle-mediated transport in synapse |
|  |  |  |  | BP | 1.23E-03 | neurotransmitter secretion |
|  |  |  |  | BP | 1.23E-03 | signal release from synapse |
| DOID:0050692 | Brody myopathy | CP;HPS3 | 29 | Not Found | | |
| DOID:0050692 | Brody myopathy | FGFR3 | 30 | BP | 1.35E-04 | chondrocyte differentiation |
|  |  |  |  | BP | 2.82E-04 | cartilage development |
|  |  |  |  | BP | 6.13E-04 | skeletal system morphogenesis |
|  |  |  |  | BP | 6.42E-04 | extrinsic apoptotic signaling pathway |
|  |  |  |  | BP | 1.26E-03 | connective tissue development |
|  |  |  |  | BP | 2.00E-03 | developmental maturation |
| DOID:0050777 | Joubert syndrome | CP;HPS3 | 31 | Not Found | | |
| DOID:9119 | Acute myeloid leukemia | DNAJC5 | 32 | BP | 2.30E-04 | synaptic vesicle exocytosis |
| DOID:1184 | Nephrotic syndrome | DNAJC5 | 33 | BP | 2.98E-04 | synaptic vesicle cycle |
|  |  |  |  | BP | 5.08E-04 | vesicle-mediated transport in synapse |
|  |  |  |  | BP | 1.23E-03 | neurotransmitter secretion |
|  |  |  |  | BP | 1.23E-03 | signal release from synapse |
| DOID:0050692 | Brody myopathy | NPR2 | 34 | BP | 4.94E-05 | heart contraction |
|  |  |  |  | BP | 5.91E-05 | cardiac conduction |
|  |  |  |  | BP | 6.29E-05 | heart process |
|  |  |  |  | BP | 1.68E-04 | regulation of heart contraction |
|  |  |  |  | BP | 3.45E-04 | regulation of cardiac conduction |
|  |  |  |  | BP | 3.59E-04 | multicellular organismal signaling |
|  |  |  |  | BP | 4.68E-04 | regulation of blood circulation |
|  |  |  |  | BP | 2.00E-03 | developmental maturation |
| DOID:552 | Pneumonia | DLL3 | 35 | BP | 6.84E-06 | pattern specification process |
|  |  |  |  | BP | 6.37E-05 | anterior/posterior pattern specification |
|  |  |  |  | BP | 2.15E-04 | regionalization |
|  |  |  |  | BP | 2.93E-04 | somitogenesis |
|  |  |  |  | BP | 7.32E-04 | somite development |
|  |  |  |  | BP | 1.13E-03 | segmentation |
| DOID:13189 | Gout | ALOX12B | 36 | BP | 2.48E-04 | ceramide biosynthetic process |
|  |  |  |  | BP | 1.13E-03 | ceramide metabolic process |
|  |  |  |  | BP | 1.45E-03 | sphingolipid biosynthetic process |
|  |  |  |  | MF | 7.09E-04 | isomerase activity |
| DOID:3883 | Lynch syndrome | WDR36 | 37 | Not Found | | |
| DOID:13189 | Gout | FGFR3 | 38 | BP | 1.35E-04 | chondrocyte differentiation |
|  |  |  |  | BP | 2.82E-04 | cartilage development |
|  |  |  |  | BP | 6.13E-04 | skeletal system morphogenesis |
|  |  |  |  | BP | 6.42E-04 | extrinsic apoptotic signaling pathway |
|  |  |  |  | BP | 1.26E-03 | connective tissue development |
|  |  |  |  | BP | 2.00E-03 | developmental maturation |
| DOID:0050692 | Brody myopathy | DNAJC5 | 39 | BP | 2.30E-04 | synaptic vesicle exocytosis |
| DOID:13189 | Gout | DNAJC5 | 40 | BP | 2.98E-04 | synaptic vesicle cycle |
| DOID:3498 | Pancreatic ductal adenocarcinoma | DNAJC5 | 41 | BP | 5.08E-04 | vesicle-mediated transport in synapse |
|  |  |  |  | BP | 1.23E-03 | neurotransmitter secretion |
|  |  |  |  | BP | 1.23E-03 | signal release from synapse |
| DOID:13099 | Moyamoya disease | ALOX12B | 42 | BP | 2.48E-04 | ceramide biosynthetic process |
|  |  |  |  | BP | 1.13E-03 | ceramide metabolic process |
|  |  |  |  | BP | 1.45E-03 | sphingolipid biosynthetic process |
|  |  |  |  | MF | 7.09E-04 | isomerase activity |
| DOID:1485 | Cystic fibrosis | CDC6 | 43 | Not Found | | |
| DOID:8781 | Rubella | DNAJC5 | 44 | BP | 2.30E-04 | synaptic vesicle exocytosis |
|  |  |  |  | BP | 2.98E-04 | synaptic vesicle cycle |
|  |  |  |  | BP | 5.08E-04 | vesicle-mediated transport in synapse |
|  |  |  |  | BP | 1.23E-03 | neurotransmitter secretion |
|  |  |  |  | BP | 1.23E-03 | signal release from synapse |
| DOID:0050692 | Brody myopathy | GINS3 | 45 | Not Found | | |
| DOID:3883 | Lynch syndrome | TGFBR1 | 46 | BP | 6.84E-06 | pattern specification process |
|  |  |  |  | BP | 6.37E-05 | anterior/posterior pattern specification |
|  |  |  |  | BP | 9.22E-05 | regulation of chondrocyte differentiation |
|  |  |  |  | MF | 4.52E-04 | SMAD binding |
| DOID:552 | Pneumonia | FGFR3 | 47 | BP | 1.35E-04 | chondrocyte differentiation |
|  |  |  |  | BP | 2.82E-04 | cartilage development |
|  |  |  |  | BP | 6.13E-04 | skeletal system morphogenesis |
|  |  |  |  | BP | 6.42E-04 | extrinsic apoptotic signaling pathway |
|  |  |  |  | BP | 1.26E-03 | connective tissue development |
|  |  |  |  | BP | 2.00E-03 | developmental maturation |
| DOID:13189 | Gout | CYP4F22 | 48 | Not Found | | |
| DOID:0050692 | Brody myopathy | LMNA | 49 | BP | 1.15E-04 | striated muscle adaptation |
|  |  |  |  | BP | 2.75E-04 | muscle system process |
|  |  |  |  | BP | 2.79E-04 | endomembrane system organization |
|  |  |  |  | BP | 3.75E-04 | muscle hypertrophy in response to stress |
|  |  |  |  | BP | 3.75E-04 | cardiac muscle adaptation |
|  |  |  |  | BP | 3.75E-04 | cardiac muscle hypertrophy in response to stress |
|  |  |  |  | BP | 5.90E-04 | regulation of apoptotic signaling pathway |
|  |  |  |  | BP | 6.42E-04 | extrinsic apoptotic signaling pathway |
|  |  |  |  | BP | 8.73E-04 | regulation of extrinsic apoptotic signaling pathway |
| DOID:3498 | Pancreatic ductal adenocarcinoma | ALOX12B | 50 | BP | 2.48E-04 | ceramide biosynthetic process |
|  |  |  |  | BP | 1.13E-03 | ceramide metabolic process |
|  |  |  |  | BP | 1.45E-03 | sphingolipid biosynthetic process |
|  |  |  |  | MF | 7.09E-04 | isomerase activity |

**Supplementary Table 1.** The table shows the top 50 predictions, and the GO enrichment analysis is conducted in R.
